# *mecA* and *mecC* Positive Strains of *Staphylococcus aureus* Detected and Isolated from Raw Milk of Ecuador

**DOI:** 10.3390/antibiotics14121255

**Published:** 2025-12-12

**Authors:** Anthony Loor-Giler, Camila Sanchez-Castro, Byron Puga-Torres, Silvana Santander-Parra, Luis Nuñez

**Affiliations:** 1 Laboratorios de Investigación, Dirección general de Investigación, Universidad de las Américas (UDLA), Antigua Vía a Nayon S/N, Quito 70124, Ecuador; a.abel.loor.giler@gmail.com; 2 Facultad de Ciencias Veterinarias, Universidad de Buenos Aires, Av. Chorroarín 280, Buenos Aires 1427, Argentina; 3 Facultad de Ingeniería y Ciencias Aplicadas, Carrera de Ingeniería en Biotecnología, Universidad de Las Américas (UDLA), Antigua Vía a Nayon S/N, Quito 170124, Ecuador; camila.sanchez.castro285@hotmail.com; 4 Facultad de Medicina Veterinaria y Zootecnia, Universidad Central del Ecuador, Jerónimo Leyton S/N y Gilberto Gatto Sobral, Quito 170521, Ecuador; bpuga@uce.edu.ec; 5 Facultad de Ciencias de la Salud, Carrera de Medicina Veterinaria, Universidad de Las Américas, Antigua Vía a Nayon S/N, Quito 170124, Ecuador; silvanahsp@yahoo.com; 6 One Health Research Group, Facultad de Ciencias de la Salud, Universidad de Las Américas, Quito 170124, Ecuador

**Keywords:** *Staphylococcus aureus*, raw milk, food contamination, infection risk

## Abstract

**Background**: Milk is a highly nutritious food, but its composition makes it an ideal medium for microbial growth, particularly for bacteria like *Staphylococcus aureus* (*S. aureus*). In Ecuador, raw milk consumption is culturally rooted, and contamination risks are heightened, especially in informal markets. *Staphylococcus aureus*, a Gram-positive, coagulase-positive bacterium, commonly colonizes mucous membranes and can cause a range of infections due to its production of thermostable toxins. Its impact extends to bovine mastitis, severely affecting dairy production. Of particular concern is the emergence of methicillin-resistant *S. aureus* (MRSA) strains, associated with the acquisition of the *mecA* gene located on the “staphylococcal chromosomal cassette mec” (SCCmec) element and identification of a *mecA* homologue, *mecC*, further complicates detection and monitoring efforts. **Objectives**: This study evaluated the prevalence of *S. aureus* and MRSA strains in raw milk from Ecuadorian provinces Pichincha and Manabí. **Methods**: A total of 633 samples were collected and analyzed via real-time PCR (qPCR) and bacterial isolation methods, complemented by endpoint PCR assays for *mecA* and *mecC* genes detection. **Results**: A high prevalence of *S. aureus* (84%) was observed, with significant differences between regions. MRSA was detected in 23% of all samples, with *mecA* being more prevalent than *mecC* among isolates. Sequencing of *16S rDNA* confirmed the identity of isolates, while phylogenetic analysis of *mecA* and *mecC* genes validated their presence. The findings suggest that suboptimal hygiene practices and varied biosecurity protocols, especially among small and medium dairy producers, may contribute to the persistence of resistant strains. **Conclusions**: This study highlights the presence of *S. aureus* and MRSA in raw milk, underscoring the need for strengthened surveillance, improved hygiene practices, the use of molecular diagnostic tools, and proper heat treatments to reduce the public health risks associated with contaminated milk and its derivatives.

## 1. Introduction

Milk is a highly nutritious food, which, due to its composition rich in minerals, vitamins, proteins and fats, serves as an essential food component for those sectors of the population with little access to other resources [1]. In Ecuador, the consumption of raw milk is deeply entrenched in the cultural fabric, particularly within rural communities where informal trade networks play a significant role, where at least 2.6 million liters of milk are consumed per year [2]. Due to its content, raw milk is an ideal medium for the growth of microorganisms such as bacteria, fungi and yeasts, where bacteria are the most common due to their high growth rate [3]. Among the most commonly found pathogenic bacteria in raw milk, *Staphylococcus aureus* (*S. aureus*) stands out due to the possible pathogenic effects on animals and humans caused by its virulence factors [4].

*S. aureus* is a spherical, Gram-positive, coagulase-positive bacterium belonging to the genus *Staphylococcus*, and is part of the microbiota of the mucous membranes, including healthy noses and viscera, of many animals [4,5]. Due to its ability to produce thermostable toxins, it can cause a wide variety of skin infections or more serious infections such as pneumonia, sepsis, endocarditis and toxic shock syndrome [6,7,8]. In cattle, *S. aureus* is one of the main causes of mastitis, both clinical and subclinical, being a problem for milk production, since it can cause permanent damage to mammary tissue [5,9]. When transmitted to humans, *S. aureus* can cause a wide range of infections, especially in moments of low immune response, where the high variety of toxins and its increasing resistance to antibiotics, especially methicillin-resistant *Staphylococcus aureus* (MRSA) strains, represent a high risk to public health [10,11].

The development of methicillin resistance is due to the acquisition of the *mecA* gene, which is located in the mobile genetic element called the “staphylococcal chromosomal cassette mec” (SCCmec) [12]. The *mecA* gene encodes penicillin-binding protein 2a (PBP2a), an enzyme involved in the synthesis of cell wall peptidoglycan, which has a low affinity for β-lactams, ensuring cell wall integrity in these strains in the presence of these antibiotics [13,14]. In 2011 in the UK, a variant of *mecA* with 70% nucleotide similarity was identified in cattle samples, subsequently named *mecC* [15]. While *mecA* is mostly found in human and domestic animal isolates, *mecC* is more common in wild animal and bovine isolates, although it has a lower global distribution than *mecA* [13]. The difference between these two genes showed that most assays designed for *mecA* are not able to detect *mecC* so differential identification of these two genes is necessary [15,16].

The prevalence of MRSA strains in food, particularly in raw milk and dairy products has been documented worldwide with varying prevalences according to region [17]. In Algeria, a 2021 study reported a 23% prevalence of MRSA strains in dairy products, along with the detection of other enterotoxin genes [18]. In Italy, in 2015, 20% of *S. aureus* isolates from raw milk were found to be MRSA [19]. In the United States in 2021, a multi-regional study of 189 farms found that 62.4% had *S. aureus* but only 0.8% were MRSA [20]. In Brazil in 2016, 23.3% of *S. aureus* strains isolated from cattle corresponded to MRSA [21]. Finally, in Colombia, a country bordering Ecuador, a prevalence of up to 47% has been reported; in addition to being elevated, it was identified that 27% of the strains contained the *mecA* gene [22]. Although *S. aureus* is usually found in milk in at least 68.8% of investigations, for the most part the presence of MRSA is usually less than 5% and increases in this rate are related to poor husbandry practices [17]. However, there is limited information regarding the presence of *mecA*- and *mecC*-carrying strains in raw milk in Ecuador, and studies addressing *mecC* in South America are particularly scarce [15,23]. Considering that raw milk consumption is still common in rural and peri-urban areas, detecting these resistance determinants is relevant for both antimicrobial resistance (AMR) surveillance and dairy safety [24,25]. Therefore, identifying the occurrence of *mecA* and *mecC* in raw milk contributes to filling a regional knowledge gap and provides evidence that may support improvements in milk hygiene practices and public health monitoring frameworks under a One Health approach [17,23].

The use of molecular techniques for the identification of contaminants is presented as an effective way, with high sensitivity and specificity for the detection of contaminants in food as well as factors that position them as risk agents for public health [26,27,28]. The aim of this study is to ascertain the prevalence of *S. aureus* in raw bovine milk from two provinces of Ecuador (Pichincha and Manabí). The study will also detect the *mecA* and *mecC* genes associated with MRSA strains, and validate a qPCR assay for their molecular identification.

## 2. Results

### 2.1. Standard Curve and Sensitivity

The dilution run in base 10 resulted in a standard curve with 98.15% efficiency and a correlation coefficient of 0.998 (Supplementary Materials). Furthermore, a limit of detection (LoD) and limit of quantification (LoQ) of up to 1 copy of genetic material was determined.

### 2.2. Prevalence and Distribution of S. aureus in Raw Milk Samples

Based on qPCR results from pre-enriched milk samples, 531 of 633 samples were positive for *S. aureus* (84.0%) (Table 1). When comparing provinces, Pichincha accounted for 303 positive samples out of 322 analyzed (94.1%), representing 57.0% (303/531) of all positive detections. In contrast, Manabí presented 228 positive samples out of 311 (73.3%), and the difference in prevalence between provinces was statistically significant (*p* < 0.0001). When evaluating producer scale, small-scale producers showed 203 positive samples out of 266 (76.3%), corresponding to 38.2% (203/531) of all positives. Medium-scale producers had 187 positives out of 232 samples (80.6%), and large-scale producers had 141 positives out of 135 samples analyzed (65.6%), without statistically significant differences among these groups (*p* > 0.05). Considering both geographic origin and production scale, small-scale producers in Pichincha had the highest number of positive samples (119/127; 93.7%), although this value did not differ significantly from medium and large producers within the same province (*p* > 0.05).

### 2.3. Isolation of Staphylococcus aureus and MRSA Strains Identification

**Figure 1:**
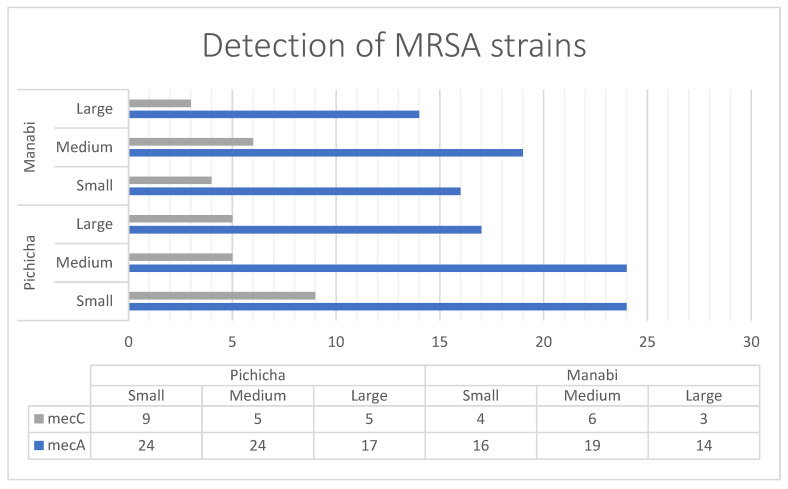
Detection and distribution of *mecA* and *mecC* positives strains of *S. aureus* based on locality and producer size.

From the samples that tested positive for *S. aureus* by qPCR, the bacterium was successfully isolated in 89.83% of cases (Table 1), confirming a strong agreement between molecular detection and culture. The concordance between both methods was supported by a Cohen’s kappa coefficient of 0.74, indicating substantial agreement. Among the isolates, 146 out of 476 tested (30.61%) carried either *mecA* or *mecC*, corresponding to an overall MRSA prevalence of 23% across all raw milk samples analyzed. The *mecA* gene was more frequently detected (114 isolates) compared to *mecC* (32 isolates). The distribution of MRSA reflected the same regional pattern observed for *S. aureus*: in Pichincha, small-scale producers showed the highest number of *mecA* and *mecC* positive isolates, while in Manabí, the highest frequency of methicillin-resistant isolates occurred among medium-scale producers, despite small producers in Manabí having a greater number of *S. aureus*-positive samples overall (Figure 1).

### 2.4. 16S Sequences Analysis

**Figure 2:**
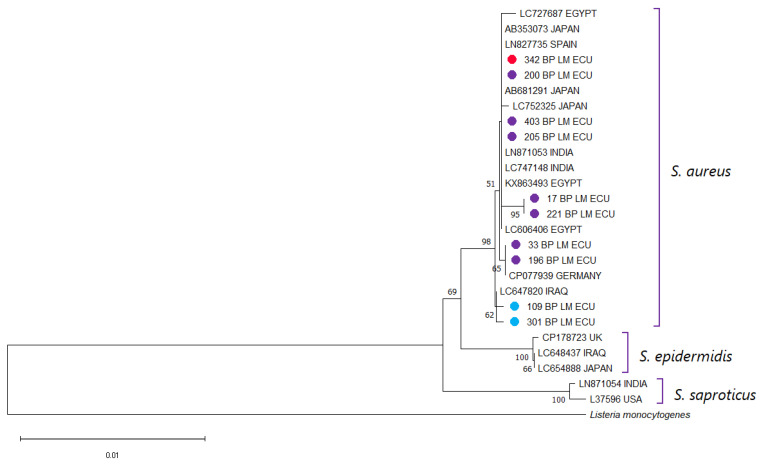
Phylogenetic analysis was performed using the partial *16S rDNA* sequences of the *S. aureus* isolates from this study together with reference sequences retrieved from NCBI. Multiple sequence alignment was carried out with Clustal X v2.0, and a phylogenetic tree was generated in MEGA 11. Bootstrap support values (1000 replicates) are indicated at the nodes, and the scale bar denotes the number of substitutions per nucleotide site. A *16S* sequence of *Listeria monocytogenes* was used as an outgroup. Sequences obtained in the present work are labeled with ● for clear identification. Isolates harboring *mecA* are displayed in red, those positive for *mecC* in blue, and non-MRSA strains in purple.

The 16S rDNA sequences obtained from the selected isolates showed >99% nucleotide identity with *S. aureus* reference sequences deposited in GenBank, confirming the taxonomic identity of the isolates. The phylogenetic tree constructed using these sequences and representative Staphylococcus spp. from GenBank grouped the isolates within the expected *S. aureus* clade, with clear separation from other species of the genus (Figure 2). As expected for a highly conserved gene such as 16S rDNA, no distinct subclustering patterns were observed among isolates carrying *mecA* or *mecC* compared to those lacking these genes. This result is consistent with the known limited ability of the 16S locus to resolve strain-level or gene-associated variation within *S. aureus*. Therefore, the 16S analysis served specifically to confirm species identity rather than to infer evolutionary relationships among isolates.

### 2.5. mecA and mecC Sequences Analysis

**Figure 3:**
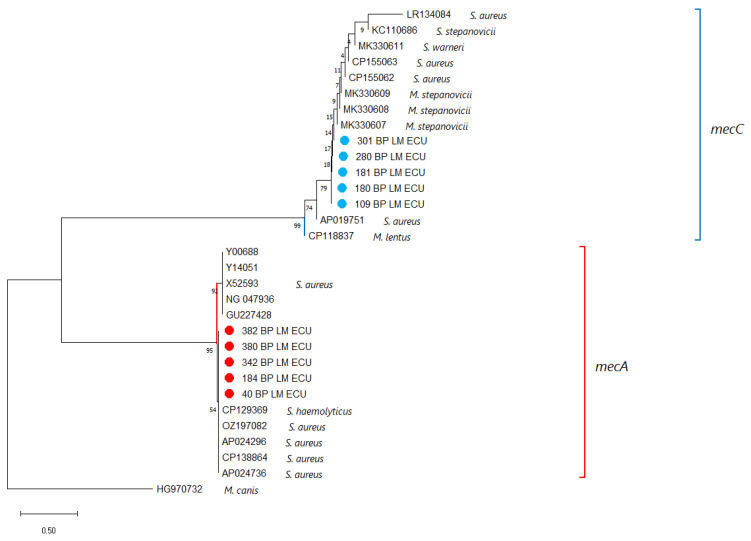
A phylogenetic tree was generated using partial nucleotide sequences of the *mecA* and *mecC* genes from this study together with homologous sequences retrieved from NCBI. Sequence alignment was performed with Clustal X v2.0, and tree reconstruction was conducted in MEGA X. Bootstrap support values, calculated from 1000 replicates, are indicated at the nodes, and the scale bar shows the number of substitutions per site. A *mecB* sequence from *Mammaliicoccus canis* served as the outgroup. Sequences obtained here are marked with ●, with *mecA*-positive isolates displayed in red and *mecC*-positive isolates in blue.

The *mecA* and *mecC* amplicons obtained from the isolates were sequenced and compared with reference sequences available in GenBank. Both genes showed 100% nucleotide identity with previously reported sequences, confirming that the resistance determinants detected in the isolates correspond to the known variants of *mecA* and *mecC* associated with methicillin resistance in *S. aureus* (Supplementary Materials). As the sequenced regions are highly conserved, no phylogenetic subdivision or variant differentiation was observed, and the sequencing results served to validate the presence of the resistance genes rather than to infer evolutionary relationships (Figure 3).

## 3. Discussion

Due to its high nutritional content, several pathogenic microorganisms tend to proliferate in raw milk, which presents a risk to public health in those population niches where it is commonly consumed [29]. Among them, *S. aureus* is presented as a risk agent with a high contamination rate in bovine-related environments, including the udder of cows and thus the milk produced [30]. Molecular methods such as qPCR are presented as a viable way for rapid, high sensitivity and specificity molecular detection of these pathogens [31]. The identification of methicillin resistance genes *mecA* and *mecC* in *S. aureus* strains is essential to determine the risk of this pathogen as a contaminant, given the symptomatology it can cause in both cattle and humans consuming contaminated milk [32]. The importance of using specific methods for the detection of the *mecC* gene differentiated from the *mecA* gene arises from the fact that those designed for the latter do not tend to have the capacity to detect all variants [33]. This study was able to identify both the presence of *S. aureus* as a contaminant agent in raw milk by qPCR and the presence of the *mecA* gene and the *mecC* gene in different isolates. These findings suggest that the contamination detected is not only widespread, but may be associated with milking hygiene practices, equipment sanitation, or herd-level mastitis dynamics rather than sporadic contamination events [34]. Furthermore, the detection of both *mecA* and *mecC* indicates the simultaneous circulation of multiple resistance determinants, which has direct implications for antimicrobial stewardship and public health, particularly in settings where raw milk consumption is common [35]. From a One Health perspective, the concurrent detection of *S. aureus* and methicillin-resistance determinants in raw milk links animal health, on-farm practices, food safety, and community exposure, underscoring that effective mitigation requires coordinated actions across veterinary, dairy-processing, and public-health stakeholders rather than isolated, sector-specific interventions [35,36].

Of the 633 samples tested, the present study found the presence of *S. aureus* in 531 (84%), being an alarmingly high prevalence, it is a wake-up call to determine the source of the high contamination rates. The results of this study are consistent with data from a study in Italy in 2020, where 80% of milk samples showed the presence of *S. aureus* and were associated with contamination in storage tanks and milking equipment [37]. Similarly in China in 2021 a 58.1% prevalence of *S. aureus* was found in raw milk samples, where contamination was associated with unhygienic handling by farmers during milking [38]. However, the trend obtained in this work contrasts with the majority of studies where the prevalence of *S. aureus* tends to be less than 50%, indicating that the high rates in this study are a consequence of deficiencies in sanitation standards [39]. This highlights the importance of evaluating farm-level milking routines, cleaning protocols, and equipment maintenance as potential drivers of bacterial persistence in the production chain. The confirmation of the bacterial strains resulted in a correspondence of the *16S* sequences obtained in this study with those previously deposited in the GenBANK close to 100% (Supplementary Material). Although bacterial isolation, even without the use of sequencing, can result in the detection of specific bacteria such as *S. aureus*, this method is more time consuming than molecular methods and can lead to a higher risk of failure due to external factors [40]. On the other hand, the isolation data shows an interesting value, where in the samples from small and medium producers at least 90% of the positives were isolated, while in the large producers this value was below 80% (Table 1). This may be due to the fact that large producers have greater control and biosecurity measures, implying that in some cases the contamination remains at low percentages of bacterial load, making isolation more difficult [41]. The isolation protocol can be made more efficient by varying the culture media used [42]. Baird-Parker agar, employed in this study, is the most suitable medium for isolating *S. aureus* in milk due to its high specificity [43]. However, using less selective media such as Mannitol Salt Agar in parallel can enhance recovery efficiency, although it increases the risk of false positives, which require careful interpretation [44]. Additionally, the use of a qPCR-based molecular method capable of detecting as few as one copy of genetic material, as implemented in this study, reduces the likelihood of false negatives [45]. Comparable surveys in other South American dairy systems report similar challenges regarding *S. aureus* contamination, although with variability linked to milking hygiene and equipment sanitation. For example, studies in Argentina and Peru have shown that herd management and milking routines heavily influence the bacterial load and strain diversity of *S. aureus* in raw milk, emphasizing the role of farm-level practices in driving contamination dynamics [46,47]. In practical terms, these findings support incremental policy measures within Ecuador’s dairy chain—such as routine screening of bulk-tank milk for *S. aureus*/MRSA at processing collection points, strengthened hygiene standard operating procedures during milking and tank sanitation, and targeted training for small and medium producers—while reinforcing existing recommendations on heat treatment for products entering formal markets [48].

MRSA among *S. aureus* isolates was particularly high (54.07%), corresponding to an overall MRSA prevalence of 23% in raw milk samples. This prevalence exceeds that reported in other studies in South America, where MRSA rates have been below 2% in dairy, indicating a potential regional problem [17]. In terms of resistance genes, *mecA* was found more frequently than *mecC*, in line with global epidemiological trends, where *mecA* remains the predominant determinant of methicillin resistance in cattle-associated *S. aureus* [17,49]. However, the detection of *mecC* in a significant proportion of isolates (32/146) is noteworthy, as *mecC* has been increasingly identified in *S. aureus* isolates of animal origin in Europe and, to a lesser extent, in South America [50,51]. This finding raises concerns about the potential for zoonotic transmission and the emergence of poorly characterized resistance mechanisms in the region. The differences observed between scales of production, with smallholders in Pichincha and medium-sized producers in Manabí harboring a higher number of *mecA* and *mecC* positive isolates, suggest that biosecurity practices and antimicrobial use policies may vary significantly depending on farm size. Previous research has shown that small farms tend to have less stringent hygiene and antibiotic stewardship practices, which may contribute to the selection and maintenance of resistant bacterial populations [52]. In contrast, medium-sized farms may experience different selection pressures due to more intensive husbandry practices and prophylactic antibiotic use, which may explain the relatively high prevalence of MRSA despite lower detection rates of *S. aureus* [53]. These findings highlight the importance of implementing training and quality control programs adapted to the size and characteristics of dairy farms [54]. This issue is particularly critical in Ecuador, where the informal consumption of milk and dairy products—those not subjected to standardized quality and safety testing—represents a significant public health concern. These products often bypass adequate heat treatment necessary to eliminate pathogenic microorganisms, and they frequently contain elevated levels of antibiotic residues [24,55]. At a broader scale, global meta-analyses indicate that the circulation of MRSA in dairy systems associated in livestock- is shaped by a combination of local antimicrobial use policies, milking system infrastructure, and mastitis control programs, rather than by geographic region alone [56]. This suggests that the patterns observed in Ecuador likely reflect production-level factors rather than intrinsic microbial characteristics [57]. In Latin America, *mecC* remains under-characterized compared with *mecA*, partly due to historical assay bias towards *mecA* and limited routine screening; therefore, documenting *mecC* alongside *mecA* provides regionally relevant evidence that can inform assay selection in laboratories and guide future source-tracking when resources allow, without over-interpreting the present cross-sectional data [58].

Despite the valuable information obtained in this study on *S. aureus* as a common contaminant in raw milk and the detection of strains carrying the *mecA* and *mecC* genes, the precise impact of this problem, either as a cause of sporadic infections in farmers and consumers or as a factor associated with increased rates of bovine mastitis, is not yet known [59]. Further studies are needed to determine the real risk posed by these circulating strains [60]. Although partial 16S rDNA sequencing confirmed the taxonomic identity of the isolates as *S. aureus*, this marker is highly conserved and therefore provides limited discriminatory power for resolving phylogenetic structure among strains [61]. Similarly, the partial *mecA* and *mecC* sequences analyzed in this study allowed the confirmation of methicillin resistance determinants, but not enabling differentiation at the lineage or clonal complex level [62]. For this reason, only broad genetic relatedness was interpreted. Future work should incorporate sequencing of more variable genomic regions or whole-genome sequencing approaches, allowing higher-resolution phylogenomic analysis and epidemiological tracing of transmission events, particularly in the context of One Health surveillance [63,64]. At the surveillance level, integrating MRSA monitoring in raw milk into national systems would align with international reporting frameworks—WHO/GLASS for antimicrobial resistance, EFSA’s risk-based food-chain monitoring approaches in the EU context, and WOAH guidance for animal-origin AMR data—facilitating comparability and priority-setting without requiring changes to the present study design. Overall, the results of this study emphasize that both detection and control strategies must consider not only pathogen identification but also the management practices that facilitate its persistence in dairy environments [65,66,67,68].

## 4. Materials and Methods

### 4.1. Sampling

The sample size required to estimate the prevalence of *Staphylococcus aureus* in bovine raw milk from Ecuador was calculated using the formula *n* = (Z^2^ × Pexp × (1 − Pexp))/d^2^, assuming an unknown population [69]. In this equation, *n* represents the number of samples, *Pexp* the expected prevalence, *d* the desired absolute precision, and *Z* the standard normal deviate corresponding to a 95% confidence level (1.96). Following this approach, a total of 633 samples were obtained over one climatic year; 322 from the highland province of Pichincha and 311 from the coastal province of Manabí, in accordance with the NTE ISO 707 standard [70]. Farms were selected using convenience sampling based on producer availability and willingness to participate. Sampling was conducted across the full annual climatic cycle to incorporate potential seasonal variation. No prior filtering based on herd health status was applied, as the goal was to capture naturally occurring prevalence in routine production settings. Producers were classified according to the Ministry Agreement No. 095 [71]: farms with fewer than 50 head of cattle were considered “small producers,” whereas those with 50–200 heads were categorized as “medium producers” (Supplementary Materials). Raw milk samples were collected directly from the bulk cooling tank after agitation to ensure homogenization. In cases where bulk tanks were not available (small producers), milk was collected immediately after milking. Before sampling, teats were cleaned with potable water and then dried, collectors used disposable sterile gloves. Approximately 50 mL of milk was aseptically transferred into sterile screw-cap containers. Milk was collected aseptically into sterile containers, transported under refrigeration (4 °C), and processed upon arrival at the laboratories of Universidad de Las Américas (UDLA). All procedures were performed under the ethical approval of the Committee for the Care and Use of Laboratory and Domestic Animals of AGROCALIDAD (authorization #INT/DA/019 2018-01-31).

### 4.2. BHI Enrichment and DNA Extraction

The collected bovine milk samples were pre-enriched in Brain Heart Infusion (BHI) broth (D, Sparks, MD, USA; Cat. No. 237500) at a 1:9 sample-to-broth ratio in a final volume of 40 mL. The cultures were incubated at 37 °C with agitation (200 rpm) for approximately 24 h [28]. After incubation, a 1 mL aliquot of the pre-enrichment was taken and subjected to DNA extraction using a Phenol-chloroform based method, using GT reagent according to the previously described protocol [72]. The procedure involved chemical lysis, phase separation, ethanol precipitation, a 70% ethanol wash, and resuspension of the pellet in 30 µL volume with UltraPure™ DNase/RNase-Free Distilled Water (Invitrogen by Thermo Fisher Scientific, Carlsbad, CA 92008, USA). The extracted DNA was stored at −20 °C until use.

### 4.3. Bacteria Isolation

The pre-enrichments were used for the isolation of *Staphylococcus aureus*. According to ISO 6888-1:2021 standard [73], after incubation the pre-enrichment BHI samples were seeded on Baird Parker Agar plates (Becton, Dickinson and Company, Franklin Lakes, CA, USA). The inoculated plates were incubated at 37 °C for approximately 19 h; after incubation, black, shiny colonies with clear halos were identified as apparently positive for *S. aureus*. To extract DNA from the isolates, selected colonies were taken and placed in 200 µL of Tris-EDTA (TE) buffer and the Boiling method was applied [74]. This consists of a thermal cell lysis followed by centrifugation to recover the supernatant containing the bacterial DNA. The extracted DNA was stored at −20 °C until use.

### 4.4. qPCR for S. aureus Detection

For specific identification of *S. aureus,* a qPCR assay was performed using previously reported primers and the corresponding hydrolysis probe (Table 2). To determine sensitivity of assay, a standard curve was constructed. For this purpose, the target fragment was first amplified by end-point PCR using only the primers Qnuc-S and Qnuc-AS (Table 2) using the 2× Promega GoTaq Green Master Mix kit (Promega, Madison, WI, USA) according to the manufacturer’s instructions. After amplicon confirmation, an enzyme purification with ExoZap-IT kit (Applied Biosystems, Santa Clara, CA 95051, USA) was carried. The generated product was inserted into a PCR 2.1-TOPO vector, (Invitrogen, Carlsbad, CA, USA), transformed and cloned into *Escherichia coli* competent cells according to the manufacturer’s instructions. The Plasmid DNA was extracted from the bacterial culture using the PureLink Quick Plasmid Miniprep Kit (Invitrogen by Thermo Fisher Scientific Baltics UAB Vilnius, Lithuania) following the manufacturer’s instructions. This was used as the basis for standard curve construction. Using the web tool DNA copy number and dilution Calculator (Thermo Fisher Scientific) it was determined the quantity of DNA plasmid necessary to make a first dilution with a known quantity of DNA copies (10^9^ copies), then, serial dilutions in base 10 were performed resulting in a standard curve from 10^9^ copies to 1 plasmid copy numbers. The qPCR assay was performed using 2× of TaqMan™ Universal Master Mix II, with UNG (Applied Biosystems by Thermo Fisher Scientific, Carlsbad, CA, USA), 0.2 µM of each primer, 0.1 µM of hydrolysis probe (Table 2), 1 µL of extracted DNA, and made up to 10 µL volume with UltraPure™ DNase/RNase-Free Distilled Water (Thermo Fisher Scientific). The qPCR protocol was performed using the following temperature conditions: started with an initial denaturation cycle for 5 min, followed for forty cycles heated to 95 °C denaturation for 10 s, 58 °C annealing for 30 s (reading was performed at this step), and 72 °C extension for 30 s; and carried out on the CFX96 Touch Real-Time PCR Detection System (Bio Rad Laboratories, Inc., Hercules, CA, USA). All DNA extracted from pre-enriched samples and isolates were subjected to this qPCR assay in duplicate, incorporating ddH_2_O as the negative control, dilutions of the previously generated curve as positive control, and a non-template control.

### 4.5. Identification of mecA and mecC Genes

To detect methicillin resistance, end-point PCR assays targeting the *mecA* and *mecC* genes were performed using the primer pairs listed in Table 2. Reactions were prepared using 2× GoTaq Green Master Mix (Promega, Madison, WI, USA), 0.3 µM of each primer, 1 µL of extracted DNA, and UltraPure™ DNase/RNase-free distilled water to a final volume of 10 µL. PCR amplification was carried out following previously reported thermal cycling parameters, and amplification products were separated by electrophoresis and stained using SYBR^®^ Safe DNA gel stain (Thermo Fisher Scientific). Amplicon sizes of 155 bp (*mecA*) and 106 bp (*mecC*) were verified using the TrackIt™ 50 bp DNA Ladder (Thermo Fisher Scientific). Isolates positive for either gene were classified as MRSA.

### 4.6. End Point PCR for 16S rDNA Sequencing

To confirm the identity of the isolates, a subset of 10 randomly selected samples was subjected to amplification of an approximately 1400 bp fragment of the 16S rDNA gene, following the protocol using the primers 27F and 1482R [75]. PCR reactions were performed using 2× GoTaq Green Master Mix (Promega, Madison, WI, USA), 0.3 µM of each primer (Table 2), 2 µL of template DNA, and UltraPure™ DNase/RNase-Free Distilled Water to a final volume of 25 µL. Thermocycling conditions followed standard kit specifications with an annealing step at 55 °C for 30 s. The resulting amplicons were purified using ExoZAP-IT enzyme (Thermo Fisher Scientific) and sequenced using the BigDye^®^ Terminator v3.1 Cycle Sequencing Kit, followed by capillary electrophoresis on an ABI 3500 Genetic Analyzer (Applied Biosystems), according to the manufacturer’s instructions. The raw data generated were analyzed in Geneious Prime v2024.0.7 (https://www.geneious.com) and aligned with sequences of *Staphylococcus* species previously deposited in GenBank using CLUSTAL X v2.0 [76], with which a Neighbor-Joining statistics method conduced in MEGA 11 [77], phylogenetic tree was constructed using the p-distance substitution model and phylogeny test bootstrap model with 1000 replicates. The sequences obtained were appropriately uploaded to GenBank and can be found under accession numbers PX525570-PX525578.

### 4.7. mecA and mecC Sequencing

To validate the veracity of the amplicons generated for *mecA* and *mecC*, 5 positive amplicons were randomly taken for each gene, sequenced and analyzed using the conditions previously indicated in the *16S rDNA* sequencing section.

### 4.8. Statistical Analysis

A descriptive analysis was conducted using variables such as province, producer size, qPCR results, and the presence of *mecA* and *mecC* in the samples. Data distribution was assessed using a Shapiro–Wilk test, and a Kruskal–Wallis test was performed to determine whether there were significant differences between variables. A cutoff of *p* ≤ 0.05 was used to determine statistical significance. To determine the difference between the pre-enriched milk qPCR and bacterial isolation assays, the Cohen’s *Kappa* coefficient between the *S. aureus* detection results of these two methods was calculated. The formula was used to determine the prevalence of the results obtained: Prevalence = (N° of positive cases)/(Total size of the assessed population). All analyses were conducted in RStudio software (version 4.4.0) 1 D

## 5. Conclusions

This study highlights the significant prevalence of *Staphylococcus aureus* contamination in raw milk, with a notable detection of methicillin resistance genes (*mecA* and *mecC*) among isolates. The high rates of *S. aureus* and MRSA found, particularly in small and medium-sized dairy operations, point towards deficiencies in hygiene practices and antimicrobial stewardship, which may contribute to the persistence and dissemination of resistant strains. The observed concordance between molecular detection by qPCR and conventional isolation supports the reliability of both methods, indicating that the applied diagnostic approach is appropriate for accurate pathogen monitoring. Furthermore, the detection of *mecC*—although less frequently reported in South America—underscores the need to strengthen genomic surveillance systems to prevent the silent spread of emerging resistance determinants with zoonotic potential. In view of the findings, it is recommended that priority be given to the following measures as part of a One Health public health framework: the improvement of routine monitoring programs, the reinforcement of milking hygiene, and the promotion of responsible antimicrobial use in livestock production. Future research should include phenotypic antimicrobial resistance profiling and whole-genome sequencing to determine transmission dynamics, virulence characteristics, and the potential impact of these strains on both bovine health and human consumers.

## Figures and Tables

**Table 1:** Comparison between *S. aureus* detection by qPCR in pre-enriched milk and successful bacterial isolation from positive samples. Percentages of qPCR positivity are calculated based on the total samples analyzed in each province (Pichincha: *n* = 322; Manabí: *n* = 311). Percentages of isolation refer to the proportion of qPCR-positive samples from which *S. aureus* was successfully recovered. Statistically significant differences are indicated by *.

Detection and Isolation of *S. aureus*							
Locality	Pichincha			Manabi *			Total
Size Producer	Small	Medium	Large	Small	Medium	Large	
**qPCR**	119 (36.96%)	104 (32.30%)	80 (24.84%)	84 (26.05%)	72 (23.15%)	72 (10.29%)	531
**Isolation**	108 (90.76%)	102 (98.08%)	63 (78.75%)	81 (96.43%)	67 (93.06%)	56 (77.78%)	477

**Table 2:** Primers and probes used in this study.

Name	Target	Assay	Sequence (5′→3′)	Reference
Qnuc-S	*nuc* gene	qPCR	CCTGAAGCAAGTGCATTTACGA	[45]
Qnuc-AS			CCTGAAGCAAGTGCATTTACGA	
Qnuc-P			HEX-CATCAGCATAAATATACGCTAAGCCACGTCCA-BHQ1	
mecA-F	*Methicillin resistance* gene A	End-point PCR	ATGGTATGTGGAAGTTAGATTGGGAT	[16]
mecA-R			TAATCTCATATGTGTTCCTGTATTGGC	
mecC-F	*Methicillin resistance* gene C		TGTCCCTAACAAAACACCCAAAGA	
mecC-R			TGGCTGAACCCATTTTTGATTAAC	
27-F	*16s rDNA*	PCR	AGAGTTTGATCMTGGCTCAG	[75]
1482-R			CGGTTACCTTGTTACGACTT	

## Data Availability

The original contributions presented in this study are included in the article/Supplementary Materials. Further inquiries can be directed to the corresponding author.

## Supplementary Materials

The following supporting information can be downloaded at: https://www.mdpi.com/article/10.3390/antibiotics14121255/s1, Data, Standard Curve qPCR and Matrix 16S.

## Abbreviations

The following abbreviations are used in this manuscript:

*S. aureus*	*Staphylococcus aureus*
AMR	Antimicrobial Resistance
ABI	Applied Biosystems
AGROCALIDAD	Agencia de Regulación y Control Fito y Zoosanitario (Ecuador)
BHI	Brain Heart Infusion
bp	Base pairs
BHQ1	Black Hole Quencher 1
CA	California
CFX96	CFX96 Touch Real-Time PCR System
CV	Coefficient of Variation
Cq	Quantification Cycle
ISO	International Organization for Standardization
SCCmec	Staphylococcal chromosomal cassette mec
PCR	Polymerase Chain Reaction
qPCR	Quantitative Polymerase Chain Reaction
EFSA	European Food Safety Authority
EU	European Union
EDTA	Ethylenediaminetetraacetic acid
LoD	Limit of Detection
LoQ	Limit of Quantification
mecA	Methicillin resistance gene A
mecC	Methicillin resistance gene C
MEGA	Molecular Evolutionary Genetics Analysis
MRSA	Methicillin-Resistant Staphylococcus aureus
NCBI	National Center for Biotechnology Information
NTC	Non-Template Control
PBP2a	Penicillin-Binding Protein 2a
Pexp	Expected prevalence
rpm	Revolutions per minute
Tris	Tris(hydroxymethyl)aminomethane
UDLA	Universidad de Las Américas
UK	United Kingdom
UNG	Uracil-N-glycosylase
WHO	World Health Organization
GLASS	Global Antimicrobial Resistance Surveillance System
WOAH	World Organisation for Animal Health
NGS	Next Generation Sequencing
